# Summary-data based Mendelian randomization identifies gene expression regulatory polymorphisms associated with bovine paratuberculosis by modulation of the *nuclear factor Kappa β* (*NF-κß)-*mediated inflammatory response

**DOI:** 10.1186/s12864-023-09710-w

**Published:** 2023-10-11

**Authors:** Gerard Badia-Bringué, Maria Canive, Nora Fernandez-Jimenez, José Luis Lavín, Rosa Casais, Cristina Blanco-Vázquez, Patricia Vázquez, Almudena Fernández, Jose Ramón Bilbao, Joseba M. Garrido, Ramón A. Juste, Oscar González-Recio, Marta Alonso-Hearn

**Affiliations:** 1 Department of Animal Health, NEIKER- Basque Institute for Agricultural Research and Development, Basque Research and Technology Alliance (BRTA), Derio, Bizkaia Spain; 2 Doctoral Program in Molecular Biology and Biomedicine, Universidad del País Vasco/Euskal Herriko Unibertsitatea (UPV/EHU), Leioa, Bizkaia Spain; 3 Department of Genetics, Physical Anthropology and Animal Physiology, University of the Basque Country (UPV/EHU), Biocruces-Bizkaia HRI, Leioa, Bizkaia Spain; 4 Department of Applied Mathematics, NEIKER- Basque Institute for Agricultural Research and Development, Basque Research and Technology Alliance (BRTA), Derio, Bizkaia Spain; 5 Center of Animal Biotechnology, SERIDA, Servicio Regional de Investigación y Desarrollo Agroalimentario, Deva, Asturias Spain; 6 Departamento de Mejora Genética Animal, Instituto Nacional de Investigación y Tecnología Agraria y Alimentaria, CSIC, Madrid, Spain

**Keywords:** Summary-data based Mendelian Randomization, GWAS, eQTL, Pleiotropy, Paratuberculosis, Holstein cattle, NF-κß

## Abstract

Genome-wide association studies (GWAS) have identified host genetic variants associated with paratuberculosis (PTB) susceptibility. Most of the GWAS-identified SNPs are in non-coding regions. Connecting these non-coding variants and downstream affected genes is a challenge and, up to date, only a few functional mutations or expression quantitative loci (cis-eQTLs) associated with PTB susceptibility have been identified. In the current study, the associations between imputed whole-genome sequence genotypes and whole RNA-Sequencing data from peripheral blood (PB) and ileocecal valve (ICV) samples of Spanish Holstein cows (*N* = 16) were analyzed with *TensorQTL*. This approach allowed the identification of 88 and 37 cis-eQTLs regulating the expression levels of 90 and 37 genes in PB and ICV samples, respectively (False discorey rate, FDR ≤ 0.05). Next, we applied summary-based data Mendelian randomization (SMR) to integrate the *cis-*eQTL dataset with GWAS data obtained from a cohort of 813 culled cattle that were classified according to the presence or absence of PTB-associated histopathological lesions in gut tissues. After multiple testing corrections (FDR ≤ 0.05), we identified two novel cis-eQTLs affecting the expression of the *early growth response factor 4* (*EGR4*) and the bovine *neuroblastoma breakpoint family member 6-like protein* isoform 2 (*MGC134040*) that showed pleiotropic associations with the presence of multifocal and diffuse lesions in gut tissues; *P* = 0.002 and *P* = 0.017, respectively. While *EGR4* acts as a brake on T-cell proliferation and cytokine production through interaction with the *nuclear factor Kappa β* (*NF-κß), MGC134040* is a target gene of *NF-κß*. Our findings provide a better understanding of the genetic factors influencing PTB outcomes, confirm that the multifocal lesions are localized/confined lesions that have different underlying host genetics than the diffuse lesions, and highlight regulatory SNPs and regulated-gene targets to design future functional studies.

**Supplementary Information:**

The online version contains supplementary material available at 10.1186/s12864-023-09710-w.

## Background

Bovine paratuberculosis (PTB) or Johne’s disease is caused by *Mycobacterium avium* subsp. *paratuberculosis* (MAP) [1, 2]. PTB is a granulomatous enteritis that affects ruminants worldwide and must be notified to the World Organization for Animal Health. The economic burden of PTB on the dairy industry relates mainly to decreased milk production and premature killing of infected animals [3]. Each year, an estimated US$198 million is lost due to PTB in the United States, US$364 million in the European Union, US$75 million in Germany, US$56 million in France, and US$12 million in Spain [4]. Infection usually occurs through the fecal–oral route at an early stage of life and can remain subclinical for years. In the jejunal-ileal Peyer's patches, MAP gains entry to the intestinal mucosa by interacting with M and epithelial cells [5–7]. MAP can survive within infected macrophages by inhibiting apoptosis and phagosome acidification, and by preventing the presentation of antigens to the immune system [8]. As the infection progresses, the lesions in the intestine and lymph nodes become more severe and the cellular infiltrate becomes diffuse, disrupting the mucosal structure and affecting the jejunum and ileum [9, 10]. Moreover, there is evidence suggesting that MAP infection is associated with human inflammatory bowel diseases (IBD), autoimmune diseases, as well as colorectal cancer and Alzheimer´s disease [11–13]. Colorectal cancer is a complication of two forms of idiopathic IBD; colonic Crohn´s disease (CD) and ulcerative colitis. Interestingly, MAP bacilli have been detected in the intestines of patients with CD, ulcerative colitis, and IBD-associated colorectal cancer [12–14].

Currently, there is not any effective treatment to cure bovine PTB, and the parenteral vaccination with heat-killed inactivated vaccines is not widely accepted by animal health authorities on the grounds of a slight interference with the diagnosis of bovine tuberculosis [15]. The identification and selection of animals naturally less susceptible to MAP infection or with increased resistance to PTB is important for disease control and breeding purposes. Genome-wide association studies (GWAS) have allowed the identification of single-nucleotide polymorphisms (SNPs) associated with PTB susceptibility, resistance, and tolerance using whole-genome sequence data (WGS) [16–25]. Although previous GWAS identified loci associated with MAP tissue infection assessed by PCR and culture [26–28], we provided the first comparison of the genetic variants associated with three distinct PTB-associated lesions in gut tissues (focal, multifocal, and diffuse) in Spanish Holstein cattle (*N* = 813) [19]. Most of the GWAS-identified SNPs are located in non-coding regions of the genome, including intergenic and intronic regions that are enriched in regulatory elements indicating that those variants exert their effects through the modulation of gene expression [29]. Connecting non-coding variants and downstream affected genes is a challenge and, up to date, only a few functional mutations or expression quantitative loci (eQTLs) with causative effects over PTB susceptibility or resistance have been characterized [30–32]. A common practice following GWAS is to map genes near the identified SNPs based on haplotype and linkage disequilibrium. However, this approach does not take into account that cis-eQTLs can be located within 1 Mb upstream of a gene transcription start site (TSS) and modulate the transcription of distant target genes [33]. Another approach for mapping cis-eQTLs-affected genes consists in finding statistically significant associations between gene expression levels and genetic variants within 1 Mb upstream of a TSS, commonly performed by linear regression [34].

In our previous study, *Matrix eQTL* [35] was used to integrate gene expression data that were mapped to the *Bos Taurus* reference genome UMD3.1 v87 with TopHat 2.1.1 [36], and genotypes obtained with the EuroG Medium Density (MD) Bead Chip of Illumina (54,609 SNPs). This approach allowed the identification of 192 and 48 cis-eQTLs associated with the expression of 145 and 43 genes in bovine peripheral blood (PB) and ileocecal valve (ICV) samples, respectively [30]. The association between these cis-eQTLs and PTB susceptibility was addressed by performing a case–control association analysis using the genotypes of the identified cis-eQTLs and phenotypical data from 986 culled cows. This approach allowed the identification of three cis-eQTLs associated with PTB susceptibility and with the up-regulation of the *MDS1* and *EVI1 complex* (*MECOM*), *eukaryotic elongation factor 1-α2* (*eEF1A2*), and *U1 spliceosomal* mRNA expression. Although this study was the first in providing insights into the role of cis-eQTLs in gene transcription regulation and PTB susceptibility, it was not performed at the WGS level.

Cis-eQTLs can alter mRNA expression leading to changes in the level, timing, and or localization of gene expression which can significantly cause variations in individual phenotypes. If the expression of a gene governed by a cis-eQTL influences a complex trait, such as a disease outcome, differences in gene expression levels between individuals will translate into differences in the disease outcome. Combined genetic-transcriptomic approaches like Mendelian Randomization (MR) allow the identification of cis-eQTLs that lead to manifestations of complex diseases or disease outcomes due to genetically regulated transcriptional activity [37]. In MR, a genetic variant is used to test the causative effect of an exposure (gene expression) on an outcome (disease outcome). However, phenotypes, WGS-derived genotypes, and gene expression data measured in a large sample size are rarely available. For this reason, Zhu et al. proposed a method called summary-data-based MR (SMR), which integrates summary-level data from an independent GWAS with data from an eQTL study to identify genes whose expression levels are associated with a complex trait due to pleiotropy, defined as”association between a trait and gene expression due to either pleiotropy (both gene expression and the trait are affected by the same causal variant) or causality (the effect of a causal variant on the trait is mediated by gene expression)” [38]. Identifying genes and genetic markers with causative effects over disease outcomes or resilience will help improve breeding progress in livestock.

**Fig. 1:**
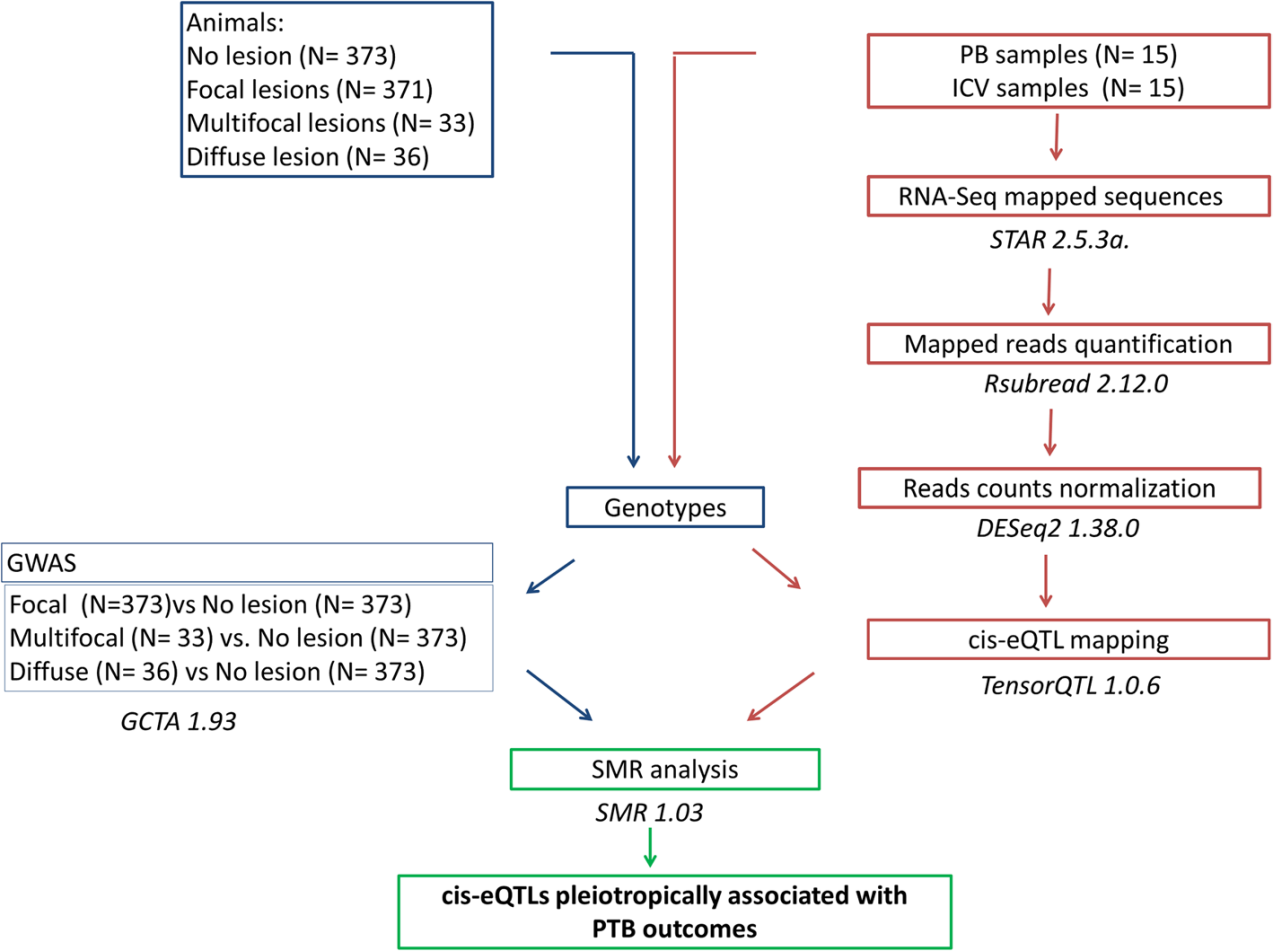
Study design. Summary data from GWAS and cis-eQTL mapping were used to perform the SMR analysis. The approach starts with the individual normalized RNA-counts, then identifies genetic variants controlling differences in gene expression, and finally checks whether the identified cis-eQTLs are indeed associated with disease outcomes using summary GWAS data from a bigger population classified according to the presence or absence of PTB-associated lesions. Two eQTL datasets from peripheral blood (PB) and ileocecal valve (ICV) were applied together with three GWAS datasets from the comparisons: focal, multifocal or diffuse lesions versus (*vs*) controls. Using these two different omics data, the SMR method discover biological mechanisms behind PTB outcomes

The main goal of this study was to identify candidate genes with pleiotropic association with cis-eQTLs and PTB-associated lesions using an SMR-based approach. First, we mapped cis-eQTLs significantly associated with gene expression changes in PB (*N* = 15) and ICV (*N* = 15) samples from 16 Holstein cows using *Tensor QTL*. For cis-eQTLs mapping, WGS-derived genotypes and RNA-Sequencing (RNA-Seq) data from ICV and PB were used [39]. Next, to test whether the pleiotropic association between these cis-eQTLs and PTB outcomes existed, an SMR analysis integrating expression data from PB and ICV datasets and three previously generated GWAS datasets from a cohort of 813 culled cows was performed. The three GWAS consisted in case–control studies in which the animals with a specific PTB-associated lesion (focal, multifocal, or diffuse) were compared with control cows without lesions in gut tissues [19]. The workflow of the study is presented in Fig. 1.

## Materials and methods

### Animals and PTB diagnosis

PB (*N* = 15) and ICV (*N* = 15) samples used in the RNA-Seq analysis were previously collected from 16 Holstein cows from a single farm in Asturias (Spain) at the time of slaughter as previously described (Table 1) [39]. Animals were 18 months old or older (mean: 4.6 years old). PB from cow ID13 and the ICV from cow ID16 were not available and were not included in the study. The infectious status of these animals was determined by histopathological analysis of gut tissues, ELISA for the detection of anti-MAP antibodies, and fecal and gut tissues PCR and bacteriological culture as previously described [40]. On the other hand, 813 culled Holstein cattle from eight different regions of Spain were used in a previous GWAS analysis [19]. Histopathological analysis of gut tissues collected from these animals was previously performed [41]. For the GWAS, cases were animals with focal (*N* = 371), multifocal (*N* = 33), or diffuse lesions (*N* = 36), while controls (*N* = 373) did not have lesions in gut tissues and had a negative ELISA, PCR, and bacteriological culture of gut tissues at the time of slaughter. The average age of the animals without lesions and with multifocal and diffuse lesions was 5.45, 5.09, and 4.38 years old, respectively.

**Table 1:** Histopathological analysis, ELISA, PCR, and bacteriological culture results from all the animals included in the current study

	**Histopathological analysis**			**ELISA**	**Fecal**	**Fecal**	**Tissue**	**Tissue**
**Animal ID**	**Microscopic**	**Macroscopic**	**ZN**		**PCR**	**Culture**	**PCR**	**Culture**
1	Negative	No	Negative	Negative	Negative	Negative	Negative	Negative
2	Negative	No	Negative	Negative	Negative	Negative	Negative	Negative
3	Negative	No	Negative	Negative	Negative	Negative	Negative	Negative
15	Negative	No	Negative	Negative	Negative	Negative	Negative	Negative
4	Focal	No	Negative	Negative	Negative	Negative	Positive	Negative
5	Focal	No	Negative	Negative	Negative	Negative	Positive	Low
6	Focal	No	Negative	Negative	Negative	Negative	Positive	Negative
7	Focal	No	Negative	Negative	Negative	Negative	Positive	Negative
8	Focal	No	Negative	Positive	Positive	Negative	Positive	Medium
9	Multifocal	Yes	Positive	Negative	Negative	Negative	Negative	Negative
16	Multifocal	No	Positive	Positive	Positive	Heavy	Positive	Heavy
10	Diffuse	Yes	Positive	Positive	Positive	Negative	Positive	Heavy
11	Diffuse	Yes	Positive	Positive	Positive	Negative	Positive	Heavy
12	Diffuse	Yes	Positive	Positive	Positive	Heavy	Positive	Heavy
13	Diffuse	Yes	Positive	Positive	Positive	Negative	Positive	Low
14	Diffuse	Yes	Positive	Positive	Positive	Negative	Positive	Heavy

Bacterial load was classified as low (< 10 CFU), medium (between 10 to 50 CFU) or heavy (> 50 CFU)

*ZN* Ziehl–Neelsen, *CFU* Colony Forming Units

### Genotyping and imputation

PB samples from the animals included in the cis-eQTL mapping and GWAS analyses were previously collected into 10 ml Vacutainer EDTA tubes (BD, Franklin Lakes, USA) for genotyping [19, 30]. Briefly, total DNA was extracted from PB samples using the QIAmp DNA Blood Mini Kit according to the manufacturer’s instructions (Qiagen, Hilden, Germany). Purified DNA was then quantified by spectrophotometry and genotyped using the EuroG medium density (MD) Bead Chip of Illumina at the molecular genetic laboratory service of the Spanish Federation of Holstein Cattle (CONAFE) using the *InfiniumTM iScan* software for allele assignation (Illumina, San Diego, USA). Individual genotypes were imputed to WGS as previously described [18]. Briefly, genotypes were phased using *Eagle 2.4* [42] and imputed with *minimac4* [43] to the Bovine High-Density Bead Chip using a reference panel of 1,278 *Bos taurus* from Run7.0 of the 1,000 Bull Genomes project and 581,712 SNPs (ARS-UCD1.2). In this step, the Run 7 population was restricted to Holstein animals. Imputation to the WGS level was then undertaken using a reference population of 2,333 *Bos taurus* from Run7.0 of the 1,000 Bull Genomes project [44]. In total, 33.77 million variants per animal, including small insertions and deletions, were obtained. SNPs with a minimum allele frequency (MAF) < 0.01 and an imputation score (R^2^) < 0.7 were excluded. After applying these two filters, all the filtered SNPs had a call rate > 95%. After applying these quality parameters, 12.38 million and 13.88 million SNPs passed the filters for the cis-eQTL mapping and GWAS populations, respectively.

### Gene expression data

Total RNA was previously isolated from PB (*N* = 15) and ICV (*N* = 15) samples of 16 Holstein cows from a single farm in Asturias (Spain) at the time of slaughter [39]. PB from cow ID13 and the ICV from cow ID16 were not available and were not included. RNA-Seq libraries were generated and then single-end sequenced in a 1 × 75 bp format using the Illumina NextSeq 500 sequencer at the Genomic Unit of the Scientific Park of Madrid, Spain. Raw reads were filtered by length (minimum size 75 bp long) and percentage of ambiguous bases less than 10% using *Prinseq-lite* 0.20.4 [45]. In the current study, reads from our previous RNA-Seq study [39] were mapped to the most recent *Bos taurus* reference genome (ARS-UCD1.2.105) with *STAR* (*Spliced Transcripts Alignment to a Reference)* 2.5.3a, an ultrafast RNA-Seq aligner capable of mapping full-length RNA sequences [46]. The reads mapped 27,607 genes. Alignment.sam files were converted to.bam files using *Samtools* 1.13 [47]. The number of reads for each gene was counted using the function “feature counts” of *Rsubread 2.12.0* [48] and normalized with the mean-of-ratios method included in the *DESeq2 1.38.0* software [49].

### Cis-eQTL mapping

For *cis*-eQTL mapping, the gene expression data (normalized counts of the 27,607 mapped genes) from the PB and ICV samples of Holstein cows from a single farm in Asturias (Spain) and corresponding WGS-derived genotypes (12,377,073 SNPs) were used to run *Tensor QTL*, which uses a fast permutation scheme that relies on the *β*-distribution to compute *P*_*β*_-values [50, 51]. Significant associations between cis-eQTLs located within 1 Mb upstream of a transcription start site (TSS) and normalized gene counts were detected. *P*_*β*_-values were corrected for multiple testing corrections with the Benjamini–Hochberg (BH) method [52] using the *R* p.adjust (pvalues, method = “fdr”) function [53]. Age was included as a covariate in the analysis.

### Genome-wide association studies (GWAS)

Associations between the imputed genotypes (13,881,067 SNPs) and the absence (*N* = 373) or presence of focal (*N* = 371), multifocal (*N* = 33), or diffuse (*N* = 36) lesions were previously analyzed in a case–control study (*N* = 813) using the mixed linear model association (mlma) analysis of the *GCTA* 1.93.2 software [19]. The model is defined as $\mathrm{y}=\mathrm{Xb}+\mathrm{g}+\mathrm{e}$ where **g** is the vector of polygenic effects and the relationship matrix is a genomic relationship matrix, with the usual notation (G) [54]. More specifically, **y** is a vector of phenotypes of length equal to n, which is the number of animals, **X** is a n x p matrix of the variables with fixed effects coded as either 0, 1 or 2, with p being the number of SNPs, **b** is a vector of the additive fixed effects of each SNP with a length of n, **g** is a vector of length n with a distribution ~ N(0,**A** ${\sigma }_{g}^{2}$), where **A** is an N x N genetic relationship matrix between individuals, and ${\sigma }_{g}^{2}$ is the variance explained by all the SNPs included in the model, and **e** is a vector of residual effects with length n with** e** ~ N(0,**I** ${\sigma }_{e}^{2}$), where **I** is an n x n identity matrix and ${\sigma }_{e}^{2}$ is any variance not explained by the SNPs included in the model [55]. Age was included as a covariate in the analysis.

### SMR analysis

Both the GWAS (.mlma file) and the cis-eQTL mapping summary data (.txt file) were used to perform the SMR analysis using the *SMR* 1.03 software [38]. Cis-eQTLs with nominal *P-*values of more than 5 × 10^–8^, and/or with differences in allele frequency between the populations of the GWAS and eQTL summary data larger than 0.2 were excluded according to Zhu et al^38^. When using SMR, specifying a threshold to remove SNPs with discrepant allele frequencies between data sets is required. That is, the SNPs with allele frequency differences between any pairwise data sets (the cis-eQTL and the GWAS summary data) large than the specified threshold (default value = 0.2) will be excluded to filter possible false positives due to allele frequency differences between the two studied populations. MR uses a cis-eQTL as a variable to estimate and test for the causative effect of an exposure variable (gene expression levels) on an outcome (presence of a specific type of PTB-associated lesion). The effect of gene expression on a specific disease outcome would then be explained by the effect of the cis-eQTL on both disease outcome and gene expression: ${\widehat{b}}_{xy}= {\widehat{b}}_{zy}/{\widehat{b}}_{zx}$ where *z* is the SNP, *x* a gene’s expression level, *y* the phenotype, ${\widehat{b}}_{zy}$ and ${\widehat{b}}_{zx}$ are the least-square estimates of *y* and *x* on *z*, respectively, and ${\widehat{b}}_{xy}$ is interpreted as the effect size of *x* on *y* free of confounding from non-genetic factors [56]. The statistic that tests for pleiotropic association (T_SMR_) would be then calculated as follows:

$${T}_{SMR}= {b}_{xy}^{2}/var\left({b}_{xy}\right)= \frac{{z}_{zy}^{2} {z}_{zx}^{2}}{{z}_{zy}^{2}+ {z}_{zx}^{2}}$$

where *z*_*zy*_ and *z*_*zx*_ being the statistics of the GWAS and eQTL analyses, respectively. *P*-values were corrected with the BH method and filtered by FDR ≤ 0.05 using R [53]. Finally, to correct for linkage disequilibrium (LD), an r^2^ threshold (default value r^2^ > 0.9) was used to remove SNPs in very strong LD with the top associated cis-eQTLs.

## Results

### RNA-Seq databases and WGS-derived genotypes

Holstein cattle (*N* = 16) included in the RNA-Seq study were tested by ELISA for the detection of MAP-specific antibodies, real-time quantitative PCR (qPCR) for the detection of MAP DNA in feces and gut issues, bacteriological culture of feces and gut tissues, and histopathological analysis, as previously described (Table 1) [39]. PB from cow ID13 and the ICV from cow ID16 were not available and were not included in the study. All control animals (*N* = 4) were negative for all the tests. Total RNA was isolated from 15 PB and 15 ICV samples, RNA-Seq libraries were generated and sequenced. In the current study, the RNA-Seq reads were mapped to the most recent *Bos taurus* reference genome (ARS-UCD1.2.105) with *STAR* 2.5.3a [46]. The number of total reads and mapped reads per individual RNA-Seq library is provided in Supplementary Table 1. Alignments of the RNA-Seq reads to the *Bos taurus* reference genome yielded mean values per library of 21.14 million reads. From the mapped reads, an average of 5% of the reads was mapped to multiple locations in the genome and was excluded. The number of mapped reads for each sample was counted and normalized using *Rsubread 2.12.0* [48] and *DESeq2* [49], respectively. On the other hand, DNA from PB samples of the 16 animals was genotyped using the Illumina EuroG MD Bead Chip (54,609 SNPs), imputed to the Bovine HD Bead Chip, and then to WGS as previously described [18]. After filtering for MAF < 0.01, a total of 12,377,073 remained.

### *Cis*-eQTL mapping

**Fig. 2:**
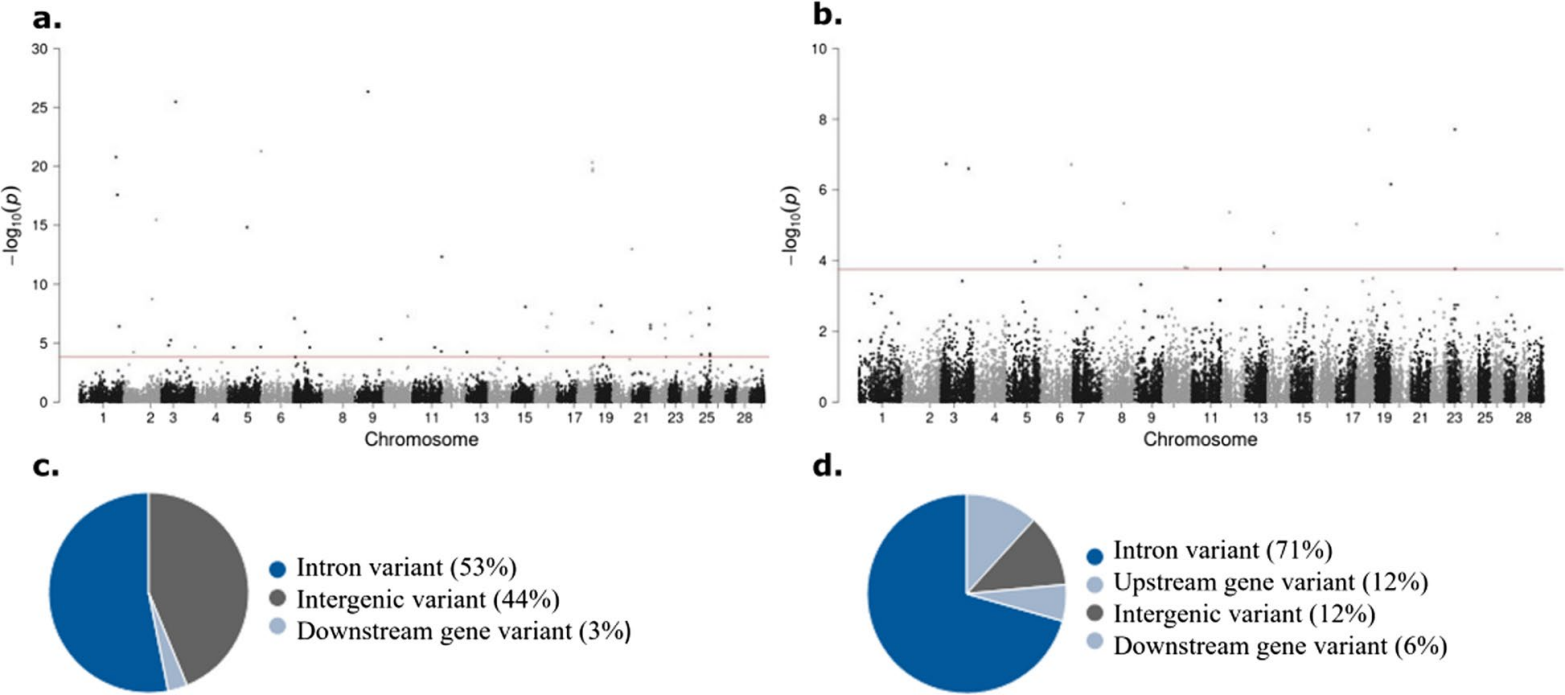
Manhattan plots of cis-eQTL mapping results. **a** cis-eQTLs identified in PB samples within 1 Mb upstream of a TSS. **b** cis-eQTLs identified in ICV samples within 1 Mb upstream of a TSS. The plot shows in the Y axis the –log_10_ (*P*_*β*_-values) of each SNP and in the X axis the chromosome where each cis-eQTL is located. Each dot represents a SNP along the *Bos taurus* genome. The dotted lines represent the *P*_*β*_-values that correspond to a FDR equal to 0.05. **c**, **d** The chart shows the genomic distributions of the PB (**c**) and ICV (**d**) cis-eQTLs identified within 1 Mb upstream of a TSS according to the Ensembl Variant Effector Predictor (VEP)

Significant associations (FDR ≤ 0.05) between cis-eQTLs located within 1 Mb upstream of a TSS and normalized gene counts were detected using *Tensor QTL* [51]. The cis-eQTLs identified within 1 Mb upstream of a TSS that are associated with changes in gene expression levels are presented in Fig. 2. We identified 88 cis-eQTLs associated with the expression of 90 genes in the PB samples (Fig. 2a) and 37 cis-eQTLs associated with expression changes of 37 genes in the ICV samples (Fig. 2b) (FDR ≤ 0.05). Most of the identified cis-eQTLs we located in intronic regions in the PB (53%) and ICV (71%) samples (Figs. 2c and d). These cis-eQTLs found in the TensorQTL analysis of PB and ICV samples and their targeted genes are presented in Supplementary Tables 2 and 3, respectively. The *cis*-eQTL with the lowest *P*_*β*_-values in the PB dataset (1.93 × 10^–34^) was associated with changes in the expression of the *Protein Phosphatase 1 Regulatory Subunit 3A* (*PPP1R3A*), a regulator of glucose homeostasis and lipid metabolism. Several cis-eQTLs identified in the PB samples regulate the expression of miRNAs such as *bta-mir-148b*, *bta-mir-2285CK*, *bta-mir-2291, bta-mir-2481, and bta-mir-10172.* In the ICV samples*,* the two *cis*-eQTLs with the lowest *P*_*β*_-value (3.60 × 10^–11^ and 4.47 × 10^–11^) regulate the expression of *LOC112449666* (small nucleolar RNAs *SNORA70*) and *bta-mir-2462* expression. Several *cis*-eQTLs identified in the ICV database were found to modify the expression of several small nucleolar RNAs (*SNORA63, SNORA47, SNORA116*), miRNAs (*bta-mir-126, bta-mir-2462*) and genes implicated in splicing (*U1, U5, U6*).

### Summary-based data Mendelian randomization (SMR) analysis

To perform the SMR analysis, *cis*-eQTLs identified in PB and ICV samples (*P*-value ≤ 5 × 10^–8^) and GWAS data from a previous study^19^ were used. In this GWAS, animals were categorized according to the presence or absence of focal, multifocal, and diffuse lesions. Therefore, a total of six SMR analyses were performed using the two sets of cis-eQTLs information in PB and ICV samples and the three GWAS summary statistics for the comparisons (animals with focal lesions *vs *controls, animals with multifocal lesions *vs* controls, and animals with diffuse lesions *vs* controls). For each SMR analysis performed, the FDR was determined. No significant cis-eQTL-gene expression-focal lesions relationship was found likely due to the lack of SNPs associated with the presence of focal lesions in the GWAS [19]. The top 10 SNPs identified in the SMR analysis using the *cis*-eQTL identified in PB and ICV samples (*P*-value ≤ 5 × 10^–8^) and GWAS data of the comparisons of cows with multifocal lesions *vs* controls and cows with diffuse lesions *vs* control can be found in Tables 2 and 3, respectively. Although several cis-eQTLs passed the genome-wide significant threshold in the SMR test (*P* ≤ 0.05), only two cis-eQTLs were significant after correction for multiple testing (False discovery rate, FDR ≤ 0.05). Several genes tagged by the top-associated cis-eQTLs (*P* ≤ 0.05, FDR > 0.05) were involved in splicing (*U6*), transcriptional regulation (*TCEA3, MECOM, EHF*), innate immune response (*SERPINB12, BPIFB6)*, apoptosis (*CIDEC*), blood coagulation (*Coagulation Factor F3, anticoagulant protein S),* and regulation of epithelial cells adhesion (*CLDN14*). A previously identified cis-eQTL-rs43744169, associated with the up-regulation of the *MECOM* expression and with increased risk for developing diffuse lesions in gut tissues after MAP infection^30^, was the top eQTL in the PB-SMR database; *P* = 0.0005 and FDR = 0.2 (Table 3).

**Table 2:** Top 10 most significant cis-eQTL found in the SMR analyses (*P*-value ≤ 0.05) using GWAS data from a case–control study where cows with multifocal lesions (*N* = 33) and without lesions (*N* = 373) were compared

Sample	Chr	Position	Gene ID	Gene symbol	SNP ID	SNP effect	*P*-value	FDR
ICV	23	27,146,264	ENSBTAG00000049699	U6	rs382341668	0.27394	0.00052194	0.134
	24	60,894,426	ENSBTAG00000035171	SERPINB12	rs477516898	0.13948	0.00171221	0.22
	18	63,798,265	ENSBTAG00000053810		rs432670715	0.220168	0.00404204	0.346
	5	106,389,826	ENSBTAG00000042924	SNORA70	rs464151990	-0.0624398	0.01562814	0.963
	29	1,195,578	ENSBTAG00000054019		rs379620385	0.0697581	0.02785573	0.963
	3	17,894,282	ENSBTAG00000053103		rs109701681	0.00163663	0.03640438	0.963
	3	20,098,666	ENSBTAG00000022741	MGC134040	rs478694916	0.055018	0.03735433	0.963
	14	63,608,763	ENSBTAG00000042171	SNORA70	rs441304008	-0.0235147	0.03771466	0.963
	6	94,896,092	ENSBTAG00000035776	CFAP299	rs459014612	0.0827706	0.04253706	0.963
	22	55,021,464	ENSBTAG00000043073	U6	rs443419569	0.0815626	0.04585341	0.963
PB	11	10,810,645	ENSBTAG00000024058	EGR4	rs383097118	0.222932	4.75E-06	0.002
	3	47,966,652	ENSBTAG00000007101	F3	rs380850681	0.00536243	0.00193635	0.207
	23	16,744,416	ENSBTAG00000015131	SLC29A1	rs137606813	0.00021436	0.00189969	0.207
	1	38,894,238	ENSBTAG00000023652	PROS1	rs385387727	0.00120501	0.00103787	0.207
	19	18,988,823	ENSBTAG00000008788	SLC13A2	rs478589506	0.24342	0.00511518	0.365
	22	6,288,529	ENSBTAG00000022744	TRIM71	rs136689901	0.0169698	0.00434811	0.365
	2	128,473,027	ENSBTAG00000038865	TCEA3	rs482027819	0.0143887	0.00868258	0.532
	3	94,262,264	ENSBTAG00000009674	C3H1orf185	rs466365210	0.216626	0.01357773	0.677
	1	72,577,888	ENSBTAG00000048826		rs109233921	-0.0774901	0.0142077	0.677
	5	106,654,304	ENSBTAG00000008553	B4GALNT3		-0.0420428	0.02157095	0.74

SNP effect- the effect size of a SNP on the phenotype that is mediated by gene expression.

*ICV* Ileocecal valve, *PB* Peripheral blood, *Chr* Chromosome, *SNP* single nucleotide polymorphism, *FDR* false discovery rate, *ID* identification code

**Table 3:** Top 10 most significant cis-eQTL found in the SMR analyses (*P*-value ≤ 0.05) using GWAS data from a case–control study where cows with diffuse lesions (*N* = 36) and without lesions (*N* = 373) were compared

Sample	Chr	Position	Gene ID	Gene symbol	SNP ID	SNP effect	*P*-value	FDR
ICV	3	20,098,666	ENSBTAG00000022741	MGC134040	rs478694916	0.103	7.53E-05	0.017
	10	25,670,772	ENSBTAG00000039241	OR6S1		0.14	1.85E-03	0.212
	3	114,640,987	ENSBTAG00000044472	5S_rRNA	rs469342481	0.065	9.70E-03	0.741
	3	17,181,908	ENSBTAG00000053743		rs471339029	0.108	0.016	0.754
	22	15,880,322	ENSBTAG00000007969	CIDEC	rs209078215	-0.0008	0.016	0.754
	13	73,824,843	ENSBTAG00000045630		rs801310416	0.086	0.027	0.798
	19	20,354,910	ENSBTAG00000042268	LOC112442820	rs481186610	0.07	0.029	0.798
	7	58,851,099	ENSBTAG00000033759	LOC777593	rs383285289	-0.087	0.03	0.798
	13	63,197,058	ENSBTAG00000010112	BPIFB6	rs110136678	0.001	0.031	0.798
	5	93,655,946	ENSBTAG00000052966	bta-mir-2285be	rs453650622	0.154	0.038	0.8
PB	1	97,501,420	ENSBTAG00000005871	MECOM	rs382448539	0.022	5.22E-04	0.201
	15	21,656,148	ENSBTAG00000015810	C15H11orf34	rs110448688	0.112	1.39E-03	0.267
	1	148,065,848	ENSBTAG00000030585	CLDN14		0.356	3.44E-03	0.441
	16	48,321,792	ENSBTAG00000045373	bta-mir-2320		0.108	7.63E-03	0.734
	15	64,738,340	ENSBTAG00000017150	EHF	rs876546467	0.053	9.83E-03	0.757
	13	74,882,914	ENSBTAG00000002942	SLC2A10	rs41711514	0.002	0.014	0.874
	3	47,966,652	ENSBTAG00000007101	F3	rs380850681	0.004	0.017	0.916
	9	9,801,563	ENSBTAG00000035054	COL9A1	rs435010900	0.04	0.021	0.986
	5	114,503,489	ENSBTAG00000006973	PHF21B	rs444663763	0.027	0.023	0.986
	15	21,822,607	ENSBTAG00000053780		rs109404405	0.029	0.026	0.986

SNP effect- the effect size of a SNP on the phenotype that is mediated by gene expression

*ICV* Ileocecal valve, *PB* Peripheral blood, *Chr* Chromosome, *SNP* Single nucleotide polymorphism, *FDR* False discovery rate, *ID* Identification code

**Fig. 3:**
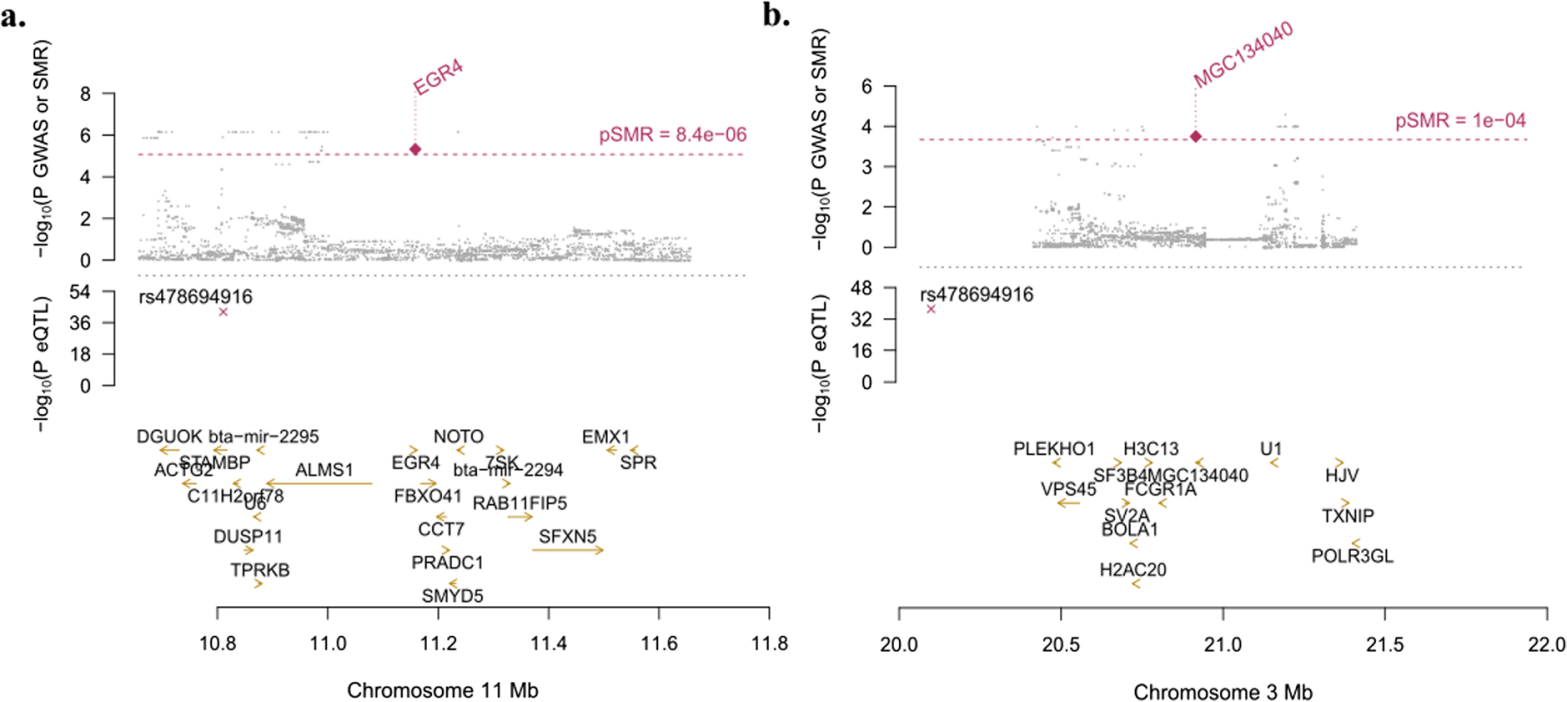
Locus plots showing the significant genes *EGR4* and *MGC134040,* their locations within chromosomes 11 and 3, respectively. Y axis represents the negative log of the significant *P*-values and X axis represents the negative log of the *P*-values. Dots in the top plots represent the *P*-values for SNPs from the GWAS analysis. Rhombuses represent the *P*-values from the SMR analyses, highlighted in red if significant. In the middle plots, crosses represent the *P*_*β*_-values of the cis-eQTLs located in the plotted region. In the bottom plots are represented all genes in each plotted region. a. SMR results using cis-eQTLs identified in PB samples and GWAs results of a case–control study where cases were animals with multifocal lesions and controls were animals without lesions. The dotted red line represents FDR ≤ 0.05 corresponding to *P* [SMR] = 10^–5^. b. SMR results using ci-eQTLs identified in ICV samples and GWAs results of a case–control study where cases were animals with diffuse lesions and controls were animals without lesions. The dotted red line represents FDR ≤ 0.05 corresponding to *P* [SMR] = 10^–4^

After multiple corrections, the *cis*-eQTL-rs383097118 (intron variant) involved in the upregulation of the *early growth response factor 4* (*EGR4*) showed a pleiotropic association with the presence of multifocal lesions (*P*_*β*_ [Tensor] = 6.47 × 10^–43^, *P* [GWAS] = 1.22 × 10^–6^, FDR [SMR] = 0.002, SNP effect [SMR] = 0.222) (Fig. 3a). Using the cis-eQTLs (*P* ≤ 5 × 10^–8^) identified in the ICV samples, we found that the *cis*-eQTL-rs478694916 (upstream gene variant) upregulated the bovine *neuroblastoma breakpoint family member 6-like protein isoform 2* (*MGC134040*) expression and showed pleiotropic association with the presence of diffuse lesions (*P*_*β*_ [Tensor] = 8.85 × 10^–38^, *P* [GWAS] = 3.16 × 10^–5^, FDR [SMR] = 0.017, SNP effect [SMR] = 0.103) (Fig. 3b).

## Discussion

Combined genetic-transcriptomic approaches allow the identification of cis-eQTLs that affect the expression levels of genes with pleiotropic effects on complex traits. In the current study, we integrated WGS-derived genotypes and RNA-Seq reads that were mapped to the most recent *Bos taurus* reference genome (ARS-UCD1.2.105) with *STAR* 2.5.3a, an ultrafast RNA-Seq aligner that exhibits better alignment precision and sensitivity than other RNA-seq aligners [46]. Using *TensorQTL* for cis-eQTL mapping, we identified 88 *cis*-eQTLs in PB and 37 *cis*-eQTLs in ICV associated with the expression of 90 and 37 genes, respectively. We recognize that the number of individual samples used in the first part of the study was small (*N* = 30). Although the number of animals used in the GWAS could be considered moderate (*N* = 813), a total of 192 and 92 SNPs defining 13 and 9 distinct QTLs were highly associated (FDR ≤ 0.05, *P* ≤ 5 × 10^−7^) with the multifocal (heritability = 0.075) and the diffuse (heritability = 0.189) lesions, respectively [19]. *cis*-eQTL data was combined with GWAS data to successfully detect two pleiotropic associations between the *EGR4* expression in PB and susceptibility to develop multifocal lesions and between the *MGC134040* expression in ICV and susceptibility to develop diffuse lesions. The list of cis-eQTLs associated with disease outcomes will be larger in the future when larger cis-eQTLs and GWAS datasets will become available. We used cis-eQTLs data as trans-eQTLs have a weaker effect size and less direct effect.

To our knowledge, this is the first study that used an SMR approach to identify cis-eQTLs regulating genes associated with PTB outcomes by pleiotropy [57]. Using this multi-omics approach to combine various sources of data, no significant cis-eQTL-gene expression-focal lesions relationship was found, probably due to the lack of discrimination between focal lesions and controls using GWAS [19]. In contrast, we found two cis-eQTLs significantly associated with the presence of multifocal and diffuse PTB-associated lesions in gut tissues. More specifically, the heterozygous (T/C) genotype in the *cis*-eQTL-rs383097118 was associated with upregulation of the *EGR4* expression and with the presence of multifocal lesions (SNP effect = 0.222). This suggests that either the variant (T/C) in the *cis*-eQTL-rs383097118 results in upregulation of *EGR4* expression and increases the susceptibility to develop multifocal lesions or that the (T/C) variant results in upregulation of *EGR4* expression, which in turn increases the susceptibility to present multifocal lesions. In other words, the presence of the heterozygous (T/C) genotype in the *cis*-eQTL-rs383097118 and the increase in *EGR4* expression were associated with the presence of multifocal lesions. *EGR4* is a key regulator of T-cell activation, differentiation, and function. MAP-antigens stimulation of T cells is induced by T-cell receptor ligation and results in the activation and the *novo* transcription of DNA-binding transcription factors including *EGR4*, *NF-κβ*, *NT-AT*, *AP-1* ad *STAT* proteins. *EGR4* is a transcription factor that contains two zinc finger domains that interact with *NF-κβ* in T-cells leading to the transcriptional control of genes encoding pro-inflammatory cytokines [58]. The absence of *EGR4* markedly enhanced the proliferation of both CD4 + and CD8 T + cells and increased the levels of *IFNɣ, IL-9, IL-10,* and *IL-21* in *EGR4* -/- mice even in the absence of stimulation [59]. These results suggest that *EGR4* induction acts as a brake on T cell proliferation and cytokine production by limiting the strength and duration of *NF-κβ* activation, making T cells poised to respond efficiently to further stimulation. In line with these findings, we hypothesize that the presence of the heterozygous genotype in the *cis*-eQTL-rs383097118 would increase *EGR4* expression and limit the *NF-κβ*-induced pro-inflammatory immune response in response to MAP infection controlling the inflammation, resulting in the induction of an anergy stage, and allowing a long-term association of MAP with the host. This will be in line with our recent findings suggesting that the multifocal lesions are localized/confined lesions that have different underlying host genetics than the diffuse lesions [19]. Similarly, it was previously demonstrated that the presence of granulomas with multifocal distribution in tuberculoid leprosy leads to the control of *Mycobacterium leprae* replication and the containment of its spread [60]. Therefore, the T/C variant in the *cis*-eQTL-rs383097118 and high *EGR4* expression could be considered markers of PTB resilience, reflecting a T-cell intrinsic property. Since it is well recognized that not all the infected animals will progress into clinical forms during their productive life, the use of genetic markers for the identification of resilient cattle might help farmers or animal health managers to select resilient and long-time asymptomatic cattle able to tolerate the disease without having their health and milk production compromised.

The heterozygous variant in the *cis*-eQTL-rs478694916 was associated with the upregulation of the bovine *MGC134040* in ICV samples and with the presence of diffuse lesions. These findings suggest that high levels of *MGC134040* are associated with PTB susceptibility, as diffuse lesions are usually a sign of clinical infection. Although there is a lack of information about the *MGC134040* gene function in cattle, *MGC134040* belongs to the *neuroblastoma breakpoint family (NBPF*), whose overexpression has been associated with cell proliferation, and with several cancers including sarcoma in humans [61]. *NBPF* members are DNA-binding transcription factors that are directly regulated by *NF-κβ * [62].

Although no other cis-eQTLs were significant after correction for multiple testing (FDR ≤ 0.05), genes tagged by significant cis-eQTLs (*P* [SMR] ≤ 0.05) were involved in splicing (*U6*), transcriptional regulation (*TCEA3, MECOM, EHF*), innate immune response (*SERPINB12, BPIFB6)*, apoptosis (*CIDEC*), blood coagulation (*Coagulation Factor F3, anticoagulant protein S),* and regulation of epithelial cells adhesion (*CLDN14*). The *cis*-eQTL-rs43744169 (T/C), associated with the upregulation of the *MECOM* and increased risk of clinical PTB in a previous study [30], was also significantly associated with an increased risk of progression to diffuse lesions by SMR (*P*[SMR] = 0.0005, FDR = 0.2). Recent findings indicated that the *MECOM* is upregulated by inflammatory stimuli, including bacteria, and that mutations in the *MECOM* make mice more susceptible to bacterial infections [63]. Allelic variants affecting the human *MECOM* have been also associated with human IBD [64]. *MECOM* is a transcriptional regulator of the *NF-κβ*-mediated inflammatory response and an oncogene whose upregulation has been associated with many types of solid cancers in humans, including colorectal cancer and acute myeloid leukemia [65, 66]*.*

Since the *NF-κβ* is a critical factor in the gut immune response against pathogens and in promoting inflammation-associated carcinomas in the gastrointestinal tract, it has been proposed that the *NF-κβ* might provide a common and critical mechanistic link between inflammation and cancer [67]. In addition, recent epidemiological and experimental studies have revealed that mycobacterium-related inflammation may be a possible mechanism of cancer pathogenesis [68]. For instance, it has been documented that *Mycobacterium tuberculosis* and non-tuberculous *Mycobacterium avium* complex infections increase the risk and mortality of pulmonary cancer, whereas *Mycobacterium ulcerans* has been correlated with skin carcinogenesis and MAP with IIBD-associated and sporadic colorectal cancer [12, 68, 69]. Our findings suggest that heterozygous variants in a specific cis-eQTL might upregulate the expression of the *MGC134040,* a transcriptional regulator of the *NF-κβ* mediated inflammatory response in macrophages and epithelial cells, causing an uncontrolled and aberrant inflammatory response which might exacerbate tissue injury in PTB-infected cattle. It has been suggested that the activation of the inflammatory response mediated by *NF-κβ* is accompanied by an increase in the proliferation rate of cells with damaged DNA which might lead to the initiation of tumorigenesis [70]. Contrarily, our study suggests that cis-eQTL-mediated upregulation of the transcriptional regulator *EGR4* might limit the *NF-κβ*-induced proinflammatory immune response to MAP infection leading to a more restricted type of lesions.

SMR allowed us to include independently collected GWAS and cis-eQTL data to enlarge the number of individuals and thus increase the statistical power. The two cis-eQTLS, rs383097118 and rs478694916, with effects on the expression of the *EGR4* and *MGC134040* genes and the resilience/susceptibility to PTB were statistically significant in the previous GWAS, but they were not among the most significant SNPs, i.e. at the top of their peaks. This is because the *Tensor QTL* algorithm only identifies cis-eQTLs within 1 Mb of a gene, so SNPs that fall out of that range will not be identified as cis-eQTLs and, therefore, the most significant variants in the GWAS might be filtered out. Further functional studies are needed to evaluate the critical significance of the identified genetic variants for diagnosis and breeding purposes and to confirm the roles of the *MGC134040* and *EGR4* in PTB susceptibility and resilience, respectively.

## Conclusions

Our results provide a better understanding of the genetic factors influencing PTB resilience and susceptibility at the whole-genome level. Using SMR we identified two novel cis-eQTLs regulating the expression levels of two transcriptional factors (*EGR4* and *MGC134040)* functionally involved in the *NF-κβ* inflammatory response. These cis-eQTLs affect the occurrence of multifocal or diffuse lesions by upregulating the expression of *EGR4* and *MGC134040*, respectively. The introduction of the cis-eQTLs identified in the current study into marker-assisted breeding programs is expected to have a relevant effect on increasing PTB natural resilience and reducing susceptibility through selection, which in turn would reduce economic losses and improve animal health. Applications of animal genetics in breeding programs are currently one of the important motors for efficient livestock production, not only to increase performance and productivity but also to ensure the resilience and health of livestock while improving longevity of animals.

### Supplementary Information

**Additional file 1: Supplementary Table I. **RNA-Seq mapped reads.

**Additional file 2: Supplementary Table 2. **Significant cis-eQTLs found in the Tensor QTL analysis of PB samples and their targeted genes.

**Additional file 3: Supplementary Table 3. **Significant cis-eQTLs found in the Tensor QTL analysis of ICV samples and their controlled genes.

## Data Availability

The dataset supporting the conclusions of this article are included within the article. RNA-Seq raw data are deposited in the NCBI Gene Expression Omnibus (GEO) database (https://www.ncbi.nlm.nih.gov/geo/) under the accession number GSE137395. Sequence data used in this study for WGS imputation are owned by the 1000 Bull Genomes Project Consortium. The sequence variants for 1800 animals from Run8 are public at the European Variation Archive under project number PRJEB42783 (https://www.ebi.ac.uk/ena/browser/view/PRJEB42783). Individual genotype data used in this study is managed by a third party, the Spanish Friesian Cattle National Federation (CONAFE). Requests for genotype data can be made to CONAFE, Ctra. de Andalucía, km. 23,600—28,340 Valdemoro, Madrid, Spain; email: conafe@conafe.com; phone: + 34 (91) 8,952,412; website: www.conafe.com. This article was submitted to an online preprint archive; https://doi.org/10.21203/rs.3.rs-2471714/v1.
